# Which growth standards should be used to identify large- and small-for-gestational age infants of mothers with type 1 diabetes? A pre-specified analysis of the CONCEPTT trial

**DOI:** 10.1186/s12884-021-03554-6

**Published:** 2021-01-29

**Authors:** Claire L. Meek, Rosa Corcoy, Elizabeth Asztalos, Laura C. Kusinski, Esther López, Denice S. Feig, Helen R. Murphy, On behalf of the CONCEPTT collaborative group, Denice S. Feig, Helen R. Murphy, Elisabeth Asztalos, Jon F. R. Barrett, Rosa Corcoy, Alberto de Leiva, Lois E. Donovan, J. Moshe Hod, Lois Jovanovic, Erin Keely, Craig Kollman, Ruth McManus, Kellie E. Murphy, Katrina Ruedy, George Tomlinson

**Affiliations:** 1 Institute of Metabolic Science, University of Cambridge, Addenbrooke’s Hospital, Box 289, Cambridge, CB2 0QQ UK; 2 Cambridge Universities NHS Foundation Trust, Cambridge, UK; 3 Servei d’Endocrinologia i Nutrició, Hospital de la Santa Creu i Sant Pau, Barcelona, Spain; 4 Institut de Recerca, Hospital de la Santa Creu i Sant Pau, CIBER-BBN, Barcelona, Spain; 5 Department de Medicina, Universitat Autònoma de Barcelona, Barcelona, Spain; 6 Sunnybrook Health Sciences Centre, University of Toronto, Toronto, Canada; 7 Servei de Pediatria, Hospital de la Santa Creu i Sant Pau, Barcelona, Spain; 8 Mount Sinai Hospital, Department of Medicine, University of Toronto, Toronto, Canada; 9 Lunenfeld-Tanenbaum Research Institute, Toronto, Canada; 10 Norwich Medical School, University of East Anglia, Norwich, UK; 11 Department of Women and Children’s Health, King’s College London, London, UK

**Keywords:** Large-for-gestational-age, Small for gestational age, Macrosomia, Birth-weight, Diabetes, Pregnancy, CONCEPTT, Growth standards, INTERGROWTH, GROW

## Abstract

**Background:**

Offspring of women with type 1 diabetes are at increased risk of fetal growth patterns which are associated with perinatal morbidity. Our aim was to compare rates of large- and small-for-gestational age (LGA; SGA) defined according to different criteria, using data from the Continuous Glucose Monitoring in Type 1 Diabetes Pregnancy Trial (CONCEPTT).

**Methods:**

This was a pre-specified analysis of CONCEPTT involving 225 pregnant women and liveborn infants from 31 international centres (ClinicalTrials.gov NCT01788527; registered 11/2/2013). Infants were weighed immediately at birth and GROW, INTERGROWTH and WHO centiles were calculated. Relative risk ratios, sensitivity and specificity were used to assess the different growth standards with respect to perinatal outcomes, including neonatal hypoglycaemia, hyperbilirubinaemia, respiratory distress, neonatal intensive care unit (NICU) admission and a composite neonatal outcome.

**Results:**

Accelerated fetal growth was common, with mean birthweight percentiles of 82.1, 85.7 and 63.9 and LGA rates of 62, 67 and 30% using GROW, INTERGROWTH and WHO standards respectively. Corresponding rates of SGA were 2.2, 1.3 and 8.9% respectively. LGA defined according to GROW centiles showed stronger associations with preterm delivery, neonatal hypoglycaemia, hyperbilirubinaemia and NICU admission. Infants born > 97.7th centile were at highest risk of complications. SGA defined according to INTERGROWTH centiles showed slightly stronger associations with perinatal outcomes.

**Conclusions:**

GROW and INTERGROWTH standards performed similarly and identified similar numbers of neonates with LGA and SGA. GROW-defined LGA and INTERGROWTH-defined SGA had slightly stronger associations with neonatal complications. WHO standards underestimated size in preterm infants and are less applicable for use in type 1 diabetes.

**Trial registration:**

This trial is registered with ClinicalTrials.gov. number NCT01788527. Trial registered 11/2/2013.

**Supplementary Information:**

The online version contains supplementary material available at 10.1186/s12884-021-03554-6.

## Background

Birth weight is an important indicator of neonatal well-being [1, 2]. Infants who are small- or large-for-gestational-age (SGA or LGA; birth weight < 10th or >90th percentile) experience higher risks of morbidity and mortality [3, 4]. Recent population based data suggests that despite improvements in care, infants of women with type 1 diabetes (T1D) remain at high risk of LGA (rates ~ 50%) [5]. LGA rates were also high in the CONCEPTT international randomized controlled trial of the use of continuous glucose monitoring (CGM) in comparison with capillary blood glucose monitoring in pregnant women with T1D [6]. LGA rates were significantly reduced in infants of women who used CGM (53% vs 69% in home blood glucose monitoring group), likely due to improved glycaemic control [7].

Currently there is controversy internationally about which growth standards to use to compare LGA rates in different populations. Customised (Gestation Related Optimum Weight; GROW) centiles [8] were used in CONCEPTT for the comparison of birthweight across international sites and diagnosis of LGA. GROW centiles are customised to maternal and neonatal factors including maternal ethnicity, height, weight, parity, neonatal sex and gestational age [7, 9]. GROW provides country-specific customised centiles, enabling international comparisons between populations. Advocates suggest that customised centiles reduce over-investigation of normal fetuses and can more accurately predict fetuses at increased risk of stillbirth and perinatal mortality [9, 10].

Other growth standards based on data from the INTERGROWTH-21st study (20,486 infants across eight geographical areas) and the World Health Organisation (WHO) Multicentre Growth Reference Study (8500 infants across six geographical areas) [11, 12] are used internationally. Both standards assume < 3.5% of the variability in growth is due to differences in ethnicity and population when circumstances are optimal (e.g healthy, well-nourished mothers) [13]. INTERGROWTH-21st standards focus on fetal growth and neonatal size at birth, while the WHO charts assess weight-for-age at 0–60 months [12]. In addition, some studies in diabetes pregnancy report standard deviation (SD) based birthweight categorisation.

Population studies comparing growth standards have focused on the identification of SGA in general maternity populations [9, 10, 14]. However, in infants of women with T1D, LGA is five times more common than in the background maternity population [5]. Identification of these infants may improve outcomes by increasing surveillance and targeting interventions to those at highest risk [15]. Furthermore, identification of a single growth standard with optimal performance in T1D pregnancies would allow future standardisation of research outcomes.

A further challenge in growth assessment in T1D pregnancy regards the use of growth standards in preterm infants. GROW and INTERGROWTH approach preterm growth differently. GROW centiles are based upon the Hadlock formula for gestational age, suggesting that the growth of preterm and term infants should be exactly the same at any timepoint [16]. The INTERGROWTH standards do not make this assumption and are based on the size of preterm infants at birth. Although this approach seems more scientifically justifiable, as growth abnormalities may contribute to preterm birth, the INTERGROWTH standards were based upon limited data from preterm (before 37 weeks) and very preterm babies (before 34 weeks), which introduces uncertainty [17]. More studies in preterm infants are needed to identify which growth standard performs best in this group [18].

Our aim was therefore to assess the incidence of LGA and SGA using different definitions and growth standards in T1D pregnancy, and to assess which standard was able to identify infants at highest risk of perinatal complications.

## Methods

The recruitment, rationale and methodology of the CONCEPTT trial are described in detail elsewhere [6] (ClinicalTrials.gov NCT01788527; registered 11/2/2013; see Appendix S1 for CONCEPTT collaborative group). In brief, women with T1D were recruited before or during pregnancy and randomized to real-time continuous glucose monitoring or capillary glucose monitoring alone. Women in the capillary glucose monitoring group also had short periods of masked continuous glucose monitoring, to allow comparison of glycaemic control between groups. Women were followed-up until delivery with collection of information about birth outcomes. Local policies in the study sites were used to determine the optimal timing and method of delivery.

Pre-specified neonatal outcomes for the CONCEPTT study included miscarriage, stillbirth, neonatal death, birth injury, shoulder dystocia, preterm delivery, neonatal hypoglycaemia requiring intravenous dextrose, hyperbilirubinaemia, respiratory distress syndrome, neonatal intensive care unit admission requiring a duration of at least 24 h, total length of hospital stay, birthweight, macrosomia (birthweight ≥4 kg), LGA (>90th centile) and SGA (< 10th centile) based on customised centiles. Definitions for CONCEPTT outcomes are given in Appendix S2 and were standardised across all the CONCEPTT sites. This data was collected using participant’s medical records. For the CONCEPTT trial, the composite neonatal endpoint incorporated pregnancy loss (miscarriage, stillbirth, or neonatal death), birth injury, neonatal hypoglycaemia, hyperbilirubinaemia, respiratory distress syndrome, or neonatal intensive care admission> 24 h. This pre-specified secondary analysis includes data from pregnant and pre-pregnant recruits who became pregnant during the 6-month pre-pregnancy trial and who gave birth to a liveborn infant. Therefore, in the current study, the composite neonatal endpoint incorporated birth injury, neonatal hypoglycaemia, hyperbilirubinaemia, respiratory distress syndrome, or neonatal intensive care admission> 24 h but not pregnancy loss.

### Calculation of birth weight centiles

Gestational age at delivery was based upon ultrasound measurements in early pregnancy (approximately 12 weeks). Maternal height and weight required for the GROW calculation was measured by trained staff at the baseline study visit. As the CONCEPTT study recruited women in early pregnancy, this was considered broadly similar to pre-pregnancy weight.

GROW centiles were calculated using version 8 (2017) of the GROW calculator using data about maternal self-reported ethnicity, parity, height, weight, gestational age at birth and neonatal sex [8]. INTERGROWTH centiles were calculated using the windows app (available at https://intergrowth21.tghn.org/intergrowth-21st-applications/; accessed 31/03/2019) using information about infant sex, weight and age (0–60 months). WHO centiles were calculated using data about infant sex, weight and age (0 months) using the igrowup package for Stata (available at http://www.who.int/childgrowth/software/en/; accessed 31/03/2019). To demonstrate differences and similarities between centile-based methods and SD-based methods in the assessment of birth weight, we also included SD-based definitions for LGA (+ 1 and 2 SDs) using methods which were commonly used in the literature. Calculation of SGA using definitions <5th and < 2.5th percentile resulted in too few infants to permit meaningful analysis.

### Statistical analysis

Continuous data were described as mean (SD) and categorical data as n (%) as appropriate. Data regarding birthweight were analysed as percentiles. An unadjusted log-binomial regression model was used to assess associations between different growth standards, at different thresholds, and perinatal outcomes. Results are presented as relative risk ratios (RR) and 95% confidence intervals. As other comparable studies in the literature provide odds ratios, we have included a table in the supplementary material with odds ratios and 95% confidence intervals, calculated using unadjusted logistic regression (Table S1). The threshold for statistical significance was *p*< 0.05. We considered that the best performing growth standard to be that which was significantly associated with the most suboptimal perinatal outcomes.

## Results

Two hundred twenty-five women and infants were included in this analysis, including 200 from the pregnancy arm and 25 from the pre-pregnancy arm who became pregnant during the trial. Baseline characteristics and pregnancy outcomes are detailed in Table 1. Most women were over 30 years old (mean age 31.4 years), overweight (mean BMI 25.8 kg/m^2^), of European or Mediterranean ethnicity (86.2%) and approximately half used insulin pump therapy (48.9%). They had T1D of 16.5 years’ duration with suboptimal glucose levels (HbA_1c_ 6.9%; 51.8 mmol/mol) in early pregnancy as defined by the trial eligibility criteria, which required HbA_1c_ >=6.5% (48 mmol/mol). Their infants were born at 37.0 weeks of gestation, predominantly by Caesarean section (68.9%).

**Table 1:** Maternal Infant Characteristics. BMI: body mass index; GROW: gestation related optimum weight; NICU: neonatal intensive care unit; WHO: World Health Organisation. Composite outcome: birth injury, neonatal hypoglycaemia, hyperbilirubinaemia, respiratory distress syndrome, or neonatal intensive care admission. Diabetes complications defined as any retinopathy, neuropathy or nephropathy

	Mean (SD) or n (%) *n*=225
MATERNAL CHARACTERISTICS	
Maternal age, years	31.4 (4.5)
BMI at enrolment, kg/m^2^	25.8 (4.6)
Ethnicity European/ Mediterranean origin	194 (86.2)
Primiparous	89 (39.6)
Duration of diabetes, years	16.5 (7.7)
Diabetes complications	58 (25.8)
Hypertension pre-pregnancy	15 (6.7)
HbA1c at randomisation mmol/mol	51.8 (6.6)
HbA1c at randomisation %	6.9 (0.6)
Smoking	20 (8.9)
Insulin pump	110 (48.9)
INFANT CHARACTERISTICS	
Sex (% male)	115 (51.1)
Gestational age at delivery	37.0 (1.6)
BIRTHWEIGHT MEASURES	
Birthweight g	3583.6 (705)
Macrosomia >=4 kg	59 (26.2) Median (range)
GROW centile	82.1 (25.9) 95.2 (0.1–100.0)
INTERGROWTH centile	85.7 (20.8) 95.0 (3.9–100.0)
WHO centile	63.9 (32.0) 73.6 (0.0–100.0)
OBSTETRIC AND PERINATAL OUTCOMES	
Caesarean section	155 (68.9)
Preterm delivery	89 (39.6)
Neonatal hypoglycaemia requiring intravenous dextrose	57 (25.3)
NICU admission	83 (36.9)
Hyperbilirubinaemia	62 (27.6)
Respiratory distress	19 (8.4)
Composite neonatal outcome	107 (47.6)

Large-for-gestational-age (LGA) rates varied (Table 2. GROW: 62.2%; INTERGROWTH 66.7%; WHO 29.8%) and there were also differences in the mean and median birthweight centile (Table 1. Mean centiles GROW: 82.1; INTERGROWTH 85.7; WHO 63.9 centiles; Median centiles GROW: 95.2; INTERGROWTH 95.0; WHO 73.6 centiles). Other measures of birth weight are shown in Table 2. Other common perinatal complications included neonatal hypoglycaemia (25.3%), hyperbilirubinaemia (27.6%), respiratory distress (8.4%) which all contributed to frequent NICU admissions > 24 h (36.9%). Birth injury and shoulder dystocia were uncommon, (1/225 (0.4%)) and only occurred in one infant who was considered LGA by all criteria. LGA according to GROW, INTERGROWTH and WHO criteria was associated with increased risks of perinatal complications (Table 2). While each growth standard was associated with some complications, no growth standard identified all complications studied. Increased birthweight according to GROW displayed more significant associations with perinatal outcomes than INTERGROWTH (i.e. with neonatal hypoglycemia, hyperbilirubinemia, NICU admission and the composite outcome).

**Table 2:** Association of adverse neonatal outcomes with low and high birthweights after GROW, INTERGROWTH and WHO criteria. Relative risk ratios and 95% confidence intervals are reported in comparison to all other pregnancies. Neonatal hypoglycaemia included only infants who required IV dextrose. GROW: gestation related optimum weight; NICU: neonatal intensive care unit; WHO: world health organisation. Composite outcome: birth injury, neonatal hypoglycaemia, hyperbilirubinaemia, respiratory distress syndrome, or neonatal intensive care admission. * *p*< 0.05; **p< 0.01; ***p< 0.001

	N (%)	Neonatal hypoglycaemia RR (95% CI)	NICU Admission RR (95% CI)	Hyper-bilirubinaemia RR (95% CI)	Respiratory Distress RR (95% CI)	Composite Outcome RR (95% CI)
**Birthweight > 84.1st centile (mean + 1 sd)**						
GROW > 84.1st centile	146/225 (64.9%)	1.66 (0.97 to 2.84)	1.41 (0.95 to 2.10)	1.56 (0.94 to 2.56)	2.03 (0.70 to 5.91)	1.46 (1.05 to 2.02)*
INTERGROWTH > 84.1st centile	167/225 (74.2%)	1.45 (0.81 to 2.61)	1.17 (0.77 to 1.77)	1.19 (0.71 to 1.99)	1.30 (0.45 to 3.77)	1.20 (0.85 to 1.69)
WHO > 84.1st centile	82/225 (36.4%)	1.36 (0.87 to 2.13)	0.99 (0.69 to 1.41)	0.77 (0.48 to 1.23)	0.80 (0.32 to 2.04)	1.00 (0.75 to 1.33)
**Birthweight >90th centile (mean + 1.28 sd)**						
GROW >90th centile	140/225 (62.2%)	1.86 (1.09 to 3.20)*	1.49 (1.01 to 2.21)*	1.61 (0.99 to 2.62)	2.28 (0.78 to 6.63)	1.56 (1.13 to 2.16)**
INTERGROWTH >90th centile	150/225 (66.7%)	1.69 (0.97 to 2.94)	1.48 (0.98 to 2.22)	1.44 (0.87 to 2.36)	1.88 (0.64 to 5.45)	1.41 (1.01 to 1.96)*
WHO >90th centile	67/225 (29.8%)	1.48 (0.94 to 2.33)	1.02 (0.70 to 1.47)	0.82 (0.50 to 1.34)	0.63 (0.22 to 1.82)	1.05 (0.78 to 1.41)
**Birthweight > 97.7th centile (mean + 2 sd)**						
GROW > 97.7th centile	95/225 (42.2%)	2.35 (1.47 to 3.75)***	1.62 (1.15 to 2.28)**	1.56 (1.02 to 2.38)*	1.88 (0.79 to 4.50)	1.50 (1.14 to 1.97)**
INTERGROWTH > 97.7th centile	92/225 (40.9%)	2.30 (1.45 to 3.65)***	1.48 (1.06 to 2.08)*	1.19 (0.78 to 1.82)	1.61 (0.68 to 3.80)	1.42 (1.08 to 1.86)*
WHO > 97.7th centile	28/225 (12.4%)	2.29 (1.45 to 3.61)***	1.55 (1.05 to 2.30)*	0.90 (0.45 to 1.77)	1.32 (0.41 to 4.24)	1.33 (0.95 to 1.86)
**Birthweight <10th centile (<1.28 sd below mean)**						
GROW <10th centile	5/225 (2.2%)	0.79 (0.13 to 4.60)	1.65 (0.79 to 3.45)	2.24 (1.06 to 4.73)*	2.44 (0.40 to 14.91)	1.27 (0.61 to 2.63)
INTERGROWTH <10th centile	3/225 (1.3%)	Insufficient events	1.83 (0.81 to 4.14)	2.47 (1.08 to 5.65)*	4.11 (0.78 to 21.63)	1.41 (0.63 to 3.18)
WHO <10th centile	20/225 (8.9%)	1.21 (0.59 to 2.45)	1.90 (1.31 to 2.77)***	1.74 (1.02 to 2.98)*	3.66 (1.47 to 9.11)**	1.67 (1.24 to 2.24)***
**Birthweight <25th centile (< 0.675 sd below mean)**						
GROW <25th centile	13/225 (5.8%)	0.59 (0.16 to 2.17)	1.05 (0.51 to 2.13)	1.43 (0.69 to 2.95)	1.92 (0.50 to 7.43)	0.80 (0.40 to 1.61)
INTERGROWTH <25th centile	7/225 (3.1%)	0.56 (0.09 to 3.46)	1.58 (0.81 to 3.07)	2.15 (1.09 to 4.23)*	3.66 (1.04 to 12.88)*	1.21 (0.63 to 2.33)
WHO <25th centile	37/225 (16.4%)	1.22 (0.70 to 2.12)	1.83 (1.31 to 2.57)***	1.77 (1.13 to 2.76)*	2.96 (1.25 to 7.02)*	1.22 (0.70 to 2.12)

**Fig. 1:**
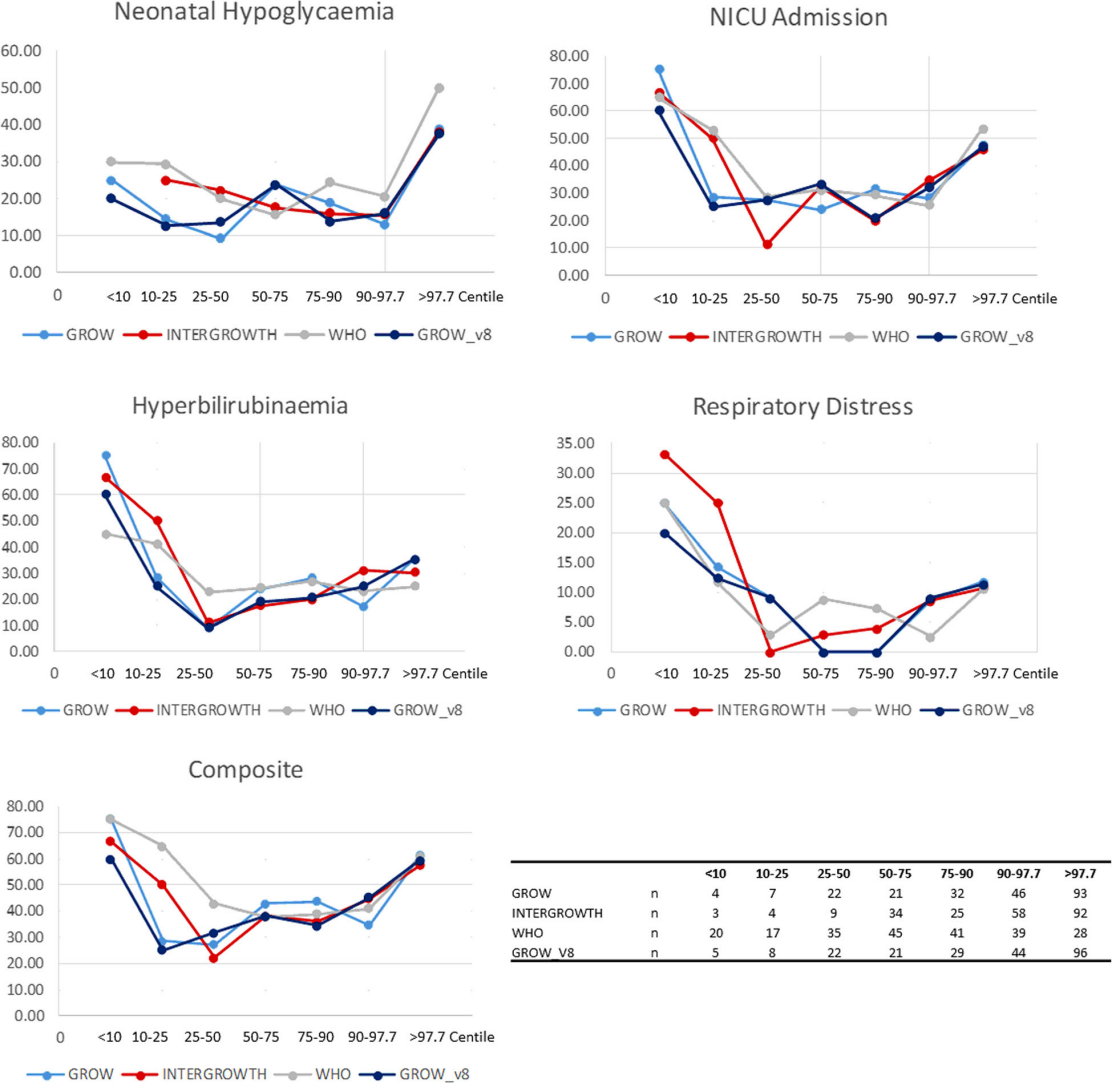
Rates (%) of caesarean delivery, preterm delivery, neonatal hypoglycaemia, hyperbilirubinaemia, respiratory distress, NICU admission and the composite neonatal outcome according to birth centile category based on GROW, INTERGROWTH and WHO standards. Numbers in each category are given at the bottom right of this figure

Most perinatal complications demonstrated a U-shaped relationship with the birth centile (Fig. 1). Neonatal hypoglycaemia was most frequent in infants born extremely large for gestational age (ELGA; > 97.7th centile). In smaller infants (<25th centile), GROW and INTERGROWTH were associated with hyperbilirubinaemia, while WHO centiles at < 10th and/or <25th centile thresholds were associated with multiple outcomes, including NICU admission, hyperbilirubinaemia, respiratory distress and the composite outcome (Table 2; Fig. 1).

Overall GROW and INTERGROWTH standards performed similarly and identified similar numbers with LGA (slightly higher for INTERGROWTH) and SGA (slightly higher for GROW). These standards performed consistently regardless of sex, ethnicity and timing of delivery (see supporting Tables S2, S3, S4). The positive and negative predictive values of the neonatal outcomes and their associated sensitivity and specificity varied depending on outcome (Table 3). The WHO standards do not take gestational age at birth into account and therefore underestimated size in preterm infants. This resulted in a linear association between WHO centile and preterm delivery (Fig. 1).

**Table 3:** Association of adverse neonatal outcomes with their positive and negative predictive values for the INTERGROWTH and WHO criteria. GROW: gestation related optimum weight; NICU: neonatal intensive care unit; WHO: world health organisation. Composite outcome: birth injury, neonatal hypoglycaemia, hyperbilirubinaemia, respiratory distress syndrome, or neonatal intensive care admission; sen: sensitivity; spe: specificity; ppv: positive predictive value; npv: negative predictive value

	Caesarean delivery				Preterm delivery				Neonatal hypoglycaemia				NICU Admission				Hyperbilirubinaemia				Respiratory Distress				Composite Outcome			
	sen	spe	ppv	npv	sen	spe	ppv	npv	sen	spe	ppv	npv	sen	spe	ppv	npv	sen	spe	ppv	npv	sen	spe	ppv	npv	sen	spe	ppv	npv
**Birthweight > 84.1st centile (mean + 1 sd)**																												
GROW > 84.1st centile	71	49	75	43	66	36	40	62	75	39	29	82	72	39	41	71	74	39	32	80	79	36	10	95	73	42	53	63
INTERGROWTH > 84.1st centile	81	40	75	48	75	26	40	62	81	28	28	81	77	27	38	67	77	27	29	76	79	26	9	93	78	29	50	59
WHO > 84.1st centile	43	77	80	38	22	54	24	52	44	66	30	78	36	63	37	63	31	61	23	70	32	63	7	91	36	64	48	52
**Birthweight >90th centile (mean + 1.28 sd)**																												
GROW >90th centile	68	51	76	42	66	40	42	65	75	42	31	84	71	43	42	72	73	42	32	80	79	39	11	95	72	47	55	65
INTERGROWTH >90th centile	72	46	75	43	66	33	39	60	77	37	29	83	75	38	41	72	74	36	31	79	79	34	10	95	74	40	53	63
WHO >90th centile	36	84	84	37	17	62	22	53	39	73	33	78	30	70	37	63	26	69	24	71	21	69	6	91	31	71	49	53
**Birthweight > 97.7th centile (mean + 2 sd)**																												
GROW > 97.7th centile	50	76	82	41	51	63	47	66	63	65	38	84	54	65	47	71	53	62	35	78	58	59	12	94	52	67	59	61
INTERGROWTH > 97.7th centile	48	76	82	40	43	60	41	62	61	66	38	83	51	65	46	69	45	61	30	74	53	60	11	93	50	67	58	59
WHO > 97.7th centile	14	90	75	32	9	85	29	59	25	92	50	78	18	91	54	65	11	87	25	72	16	88	11	92	16	91	61	54
GROW <10th centile	3	99	80	31	3	99	60	61	2	98	20	75	4	99	60	64	5	99	60	73	5	98	20	92	3	98	60	53
INTERGROWTH <10th centile	2	100	100	32	2	99	67	61	0	98	0	74	2	99	67	64	3	99	67	73	5	99	33	92	2	99	67	53
WHO <10th centile	10	94	80	32	18	97	80	64	11	92	30	75	16	95	65	66	15	93	45	74	26	93	25	93	14	96	75	55
GROW <25th centile	7	97	85	32	8	96	54	61	4	93	15	74	6	94	38	63	8	95	38	73	11	95	15	92	5	93	38	52
INTERGROWTH <25th centile	3	97	71	31	4	98	57	61	2	96	14	74	5	98	57	64	6	98	57	73	11	98	29	92	4	97	57	53
WHO <25th centile	18	87	76	32	31	93	76	68	19	85	30	76	27	89	59	68	26	87	43	76	37	85	19	94	24	91	70	57

## Discussion

This study demonstrates that GROW and INTERGROWTH growth standards perform comparably in type 1 diabetes pregnancies, giving similar median birthweight centiles and comparable rates of LGA and SGA neonates. Furthermore, both GROW and INTERGROWTH defined LGA identified neonates at increased risk of complications. LGA defined according to GROW (>90th and/or > 97.7th centile) showed stronger associations with preterm delivery, neonatal hypoglycaemia, hyperbilirubinaemia and NICU admission. In smaller infants, SGA defined according to INTERGROWTH criteria showed slightly stronger associations with outcomes. However, using any thresholds studied, weight related measures alone were not strong predictors of suboptimal perinatal outcomes (Table 3).

WHO standards do not incorporate gestational age at delivery, and thus fail to adequately describe size at birth in preterm infants. For term infants, the WHO criteria gave a true birthweight centile, but for preterm infants, the WHO criteria gave a low centile, which reflected their prematurity, not their comparative size at birth. This measure of prematurity means that the WHO criteria were still able to predict outcomes, which demonstrates the importance of preterm delivery in relation to multiple neonatal complications. However, the inability to reliably attribute a birthweight centile is a substantial limitation in type 1 diabetes pregnancies, where rates of preterm delivery are as high as 40% [5].

A central aspect to the controversy about GROW and INTERGROWTH centiles involves the perceived importance of maternal factors to the growth of the infant, and ethnicity in particular. Proponents of GROW customised centiles believe that incorporating maternal variables results in a more accurate representation of size at birth [9, 19]. Conversely, proponents of the INTERGROWTH-21st standards claim that variables such as ethnicity make little difference to size at birth, in well-nourished populations with access to adequate antenatal care [13]. A limitation of the CONCEPTT trial is that while it international, 86% of women recruited were of European / Mediterranean origin, which reduced the opportunity to look at growth standard performance in different ethnicities. A further issue is that women who choose to participate in studies are often affluent, well-nourished and educated, and may not represent mothers with different socioeconomic circumstances.

A major focus for growth standards has been on the identification of infants who are SGA with a view to reducing stillbirth rates [9]. Although we have identified that infants <25th centile displayed a trend to be at highest risk of multiple complications, very few infants fell into this category which made detailed assessment of SGA outcomes challenging. Although SGA is uncommon in type 1 diabetes pregnancy, it is plausible that infants born < 10th centile do not represent all those with growth restriction.

In this study, standard-deviation-based criteria for the diagnosis of LGA have been assessed. Although a birth weight z score > 1 is considered consistent with LGA, this definition is different to standard centile-based definitions (>90th centile) [20]. Different approaches to the LGA diagnosis contribute to difficulty in comparing populations internationally [21–23].

A fundamental aim of antenatal care in T1D pregnancies involves careful control of maternal glycaemia to normalise fetal growth. This study shows that growth –related measures alone are not strong predictors of suboptimal perinatal outcomes. For example, a birthweight >90th centile on GROW and INTERGROWTH criteria could identify neonatal hypoglycaemia, NICU admission and respiratory distress with 71–77% sensitivity, but the specificity for these outcomes was around 32–42%.

These data are consistent with other work which highlights the challenges of accurate prediction of neonatal outcomes in T1D pregnancy. For example, Yamamoto and colleagues demonstrated that LGA was the only significant predictor for neonatal hypoglycaemia on adjusted logistic regression analysis (odds ratio 2.51, 95% CI 1.10–5.70) [24]. However, 36% of infants with neonatal hypoglycaemia were appropriate for gestational age, resulting in similar levels of sensitivity and specificity seen in the current report.

As only a small proportion of the general maternity population has type 1 diabetes, CONCEPTT represents one of the larger randomised trials with detailed data on perinatal outcomes, making it useful to assess fetal growth, and infant birthweight outcomes. Customised (GROW) centiles were reported for CONCEPTT, but the effect of the intervention was also seen using INTERGROWTH standards. Although accelerated fetal growth is common in T1D pregnancies, the rates of LGA in the CONCEPTT infants were higher than expected (66% in CONCEPTT compared to ~ 50% in a UK population using similar methodology [5]). The reasons for this are unclear, particularly as the CONCEPTT population had better glycaemic control compared to the UK clinical population [5].

Despite maternal diabetes being a risk factor of perinatal morbidity, there has been relatively little assessment of different growth standards in this population. Kase and colleagues reported that customised centiles identified more infants as SGA/LGA compared to population centiles in diabetes pregnancies [25]. Narchi and Skinner had similar findings but concluded there was no evidence of a difference in mortality or morbidity between the infants identified by customised vs population growth standards [26]. The current study adds to the literature by highlighting differences between the common growth standards in a generalisable population of pregnant women with type 1 diabetes. We had no women with optimal glucose control (defined as HbA1c< 48 mmol/mol) in early pregnancy as these women were excluded from the CONCEPTT trial. However, population based studies confirm that only 15% of women with T1D achieve a first trimester HbA1c level < 6.5% (48 mmol/mol [5]) and thus the data presented here is representative for the vast majority of women. Future studies should evaluate growth standards and definitions of LGA and SGA in larger cohorts of women with diabetes, including among women with target HbA1c levels. Better understanding of the causes and early identification of growth restriction in diabetes pregnancy should be a research priority.

## Conclusions

WHO growth standards do not incorporate gestational age at birth and therefore are unsuitable for use in type 1 diabetes pregnancy, where preterm delivery is commonplace. However, GROW and INTERGROWTH standards are both suitable. Infants born > 97.7th centile had the highest risks of suboptimal outcomes. LGA defined by GROW and SGA defined by INTERGROWTH showed strongest associations with neonatal outcomes.

## Supplementary Information

**Additional file 1.**

## Data Availability

The datasets used and/or analysed during the current study are available from the corresponding author on reasonable request.

## Abbreviations

LGA: Large for gestational age
SGA: Small for gestational age
CONCEPTT: Continuous Glucose Monitoring in Type 1 Diabetes Pregnancy Trial
NICU: Neonatal intensive care unit
T1D: Type 1 diabetes
CGM: Continuous glucose monitoring
GROW: Gestation Related Optimum Weight
WHO: World Health Organisation
SD: Standard deviation
BMI: Body mass index
