# Supplementary material for: Which growth standards should be used to identify large- and small-for-gestational age infants of mothers with type 1 diabetes? A pre-specified analysis of the CONCEPTT trial
Source: BMC Pregnancy Childbirth. 2021 Jan 29;21:96. doi: 10.1186/s12884-021-03554-6 (PMC7845036; doi:10.1186/s12884-021-03554-6)
Supplement: Supplementary file 1 — Additional file 1. [file 12884_2021_3554_MOESM1_ESM.docx]

**Supplementary information**

**Table S1:** Association of adverse neonatal outcomes with low and high birthweights after GROW, INTERGROWTH and WHO criteria. Unadjusted odds ratios and 95% confidence intervals are reported in comparison to all other pregnancies. Neonatal hypoglycaemia included only infants who required IV dextrose. GROW: gestation related optimum weight; NICU: neonatal intensive care unit; WHO: world health organisation. Composite outcome: birth injury, neonatal hypoglycaemia, hyperbilirubinaemia, respiratory distress syndrome, or neonatal intensive care admission. * p<0.05; **p<0.01; ***p<0.001.

|  | **n(%)** | **Caesarean delivery**  **OR (95% CI)** | **Preterm delivery**  **OR (95% CI)** | **Neonatal hypoglycaemia**  **OR (95% CI)** | **NICU Admission**  **OR (95% CI)** | **Hyper-bilirubinaemia**  **OR (95% CI)** | **Respiratory Distress**  **OR (95% CI)** | **Composite Outcome**  **OR (95% CI)** |  |
| --- | --- | --- | --- | --- | --- | --- | --- | --- | --- |
| **Birthweight >84.1^st^ centile (mean +1 sd)** | | | | | | | |  |  |
| GROW >84.1^st^ centile | 146/225 (64.9%) | 2.31 (1.29 to 4.14)** | 1.11 (0.63 to 1.94) | 1.94 (0.98 to 3.82) | 1.70 (0.94 to 3.05) | 1.81 (0.95 to 3.47) | 2.15 (0.69 to 6.71) | 1.98 (1.13 to 3.47)* |  |
| INTERGROWTH >84.1^st^ centile | 167/225 (74.2%) | 2.78 (1.49 to 5.18)*** | 1.10 (0.59 to 2.03) | 1.62 (0.78 to 3.40) | 1.28 (0.68 to 2.40) | 1.27 (0.64 to 2.52) | 1.33 (0.42 to 4.19) | 1.40 (0.77 to 2.56) |  |
| WHO >84.1^st^ centile | 82/225 (36.4%) | 2.50 (1.32 to 4.76)** | 0.35 (0.19 to 0.63)*** | 1.52 (0.82 to 2.81) | 0.98 (0.56 to 1.72) | 0.70 (0.38 to 1.31) | 0.79 (0.29 to 2.16) | 1.00 (0.58 to 1.72) |  |
| **Birthweight >90^th^ centile (mean +1.28 sd)** | | | | | | | |  |  |
| GROW >90^th^ centile | 140/225 (62.2%) | 2.29 (1.28 to 4.08)** | 1.34 (0.76 to 2.33) | 2.25 (1.14 to 4.42)* | 1.85 (1.04 to 3.30)* | 1.89 (1.00 to 3.59)* | 2.43 (0.78 to 7.58) | 2.24 (1.29 to 3.91)** |  |
| INTERGROWTH >90^th^ centile | 150/225 (66.7%) | 2.19 (1.22 to 3.95)** | 0.97 (0.55 to 1.71) | 1.98 (0.99 to 3.96) | 1.81 (0.99 to 3.30) | 1.63 (0.85 to 3.13) | 1.97 (0.63 to 6.17) | 1.87 (1.06 to 3.29)* |  |
| WHO >90^th^ centile | 67/225 (29.8%) | 3.03 (1.47 to 6.25)** | 0.33 (0.17 to 0.63)*** | 1.72 (0.91 to 3.24) | 1.03 (0.57 to 1.85) | 0.76 (0.40 to 1.48) | 0.61 (0.19 to 1.90) | 1.10 (0.62 to 1.95) |  |
| **Birthweight >97.7^th^ centile (mean +2 sd)** | | | | | | | |  |  |
| GROW >97.7^th^ centile | 95/225 (42.2%) | 3.16 (1.68 to 5.93)*** | 1.76 (1.02 to 3.03)* | 3.17 (1.70 to 5.91)*** | 2.18 (1.25 to 3.79)** | 1.85 (1.03 to 3.35)* | 2.00 (0.77 to 5.17) | 2.22 (1.30 to 3.81)** |  |
| INTERGROWTH >97.7^th^ centile | 92/225 (40.9%) | 2.92 (1.56 to 5.49)*** | 1.13 (0.66 to 1.95) | 3.10 (1.66 to 5.77)*** | 1.88 (1.09 to 3.27)* | 1.27 (0.71 to 2.30) | 1.68 (0.65 to 4.31) | 1.99 (1.16 to 3.41)* |  |
| WHO >97.7^th^ centile | 28/225 (12.4%) | 1.41 (0.57 to 3.49) | 0.57 (0.24 to 1.36) | 3.58 (1.59 to 8.09)** | 2.19 (0.98 to 4.87) | 0.86 (0.35 to 2.14) | 1.36 (0.37 to 4.99) | 1.84 (0.82 to 4.12) |  |
| **Birthweight <10^th^ centile (<1.28 sd below mean)** | | | | | | | |  |  |
| GROW <10^th^ centile | 5/225 (2.2%) | 1.83 (0.20 to 16.66) | 2.34 (0.38 to 14.28) | 0.73 (0.08 to 6.69) | 2.63 (0.43 to 16.04) | 4.09 (0.67 to 25.11) | 2.81 (0.30 to 26.45) | 1.67 (0.27 to 10.21) |  |
| INTERGROWTH <10^th^ centile | 3/225 (1.3%) | insufficient events | 3.10 (0.28 to 34.75) | insufficient events | 3.48 (0.31 to 38.99) | 5.40 (0.48 to 60.65) | 5.67 (0.49 to 65.56) | 2.23 (0.20 to 24.93) |  |
| WHO <10^th^ centile | 20/225 (8.9%) | 1.90 (0.61 to 5.90) | 7.23 (2.33 to 22.44)*** | 1.29 (0.47 to 3.54) | 3.58 (1.37 to 9.38)** | 2.35 (0.92 to 5.98) | 4.55 (1.44 to 14.34)** | 3.68 (1.29 to 10.52)* |  |
| **Birthweight <25^th^ centile (<0.675 sd below mean)** | | | | | | | |  |  |
| GROW <25^th^ centile | 13/225 (5.8%) | 2.60 (0.56 to 12.04) | 1.85 (0.60 to 5.70) | 0.52 (0.11 to 2.42) | 1.07 (0.34 to 3.40) | 1.70 (0.53 to 5.41) | 2.09 (0.43 to 10.19) | 0.67 (0.21 to 2.13) |  |
| INTERGROWTH <25^th^ centile | 7/225 (3.1%) | 1.13 (0.21 to 5.99) | 2.09 (0.46 to 9.55) | 0.48 (0.06 to 4.09) | 2.35 (0.51 to 10.75) | 3.68 (0.80 to 16.93) | 4.73 (0.85 to 26.23) | 1.49 (0.33 to 6.81) |  |
| WHO <25^th^ centile | 37/225 (16.4%) | 1.49 (0.66 to 3.36) | 6.48 (2.88 to 14.57)*** | 1.31 (0.60 to 2.85) | 3.05 (1.48 to 6.30)** | 2.35 (1.13 to 4.88)* | 3.42 (1.25 to 9.39)* | 3.12 (1.46 to 6.69)** |  |

**Table S2:** Comparison of GROW, INTERGROWTH and WHO growth standards in infants of women of European/Mediterranean ethnicity and other ethnic groups. Data are presented as mean (SD) or n (%).

| BIRTHWEIGHT MEASURES | All infants  n=225 | Mediterranean/  European ethnicity  n=194 | Other ethnic groups  n=31 |
| --- | --- | --- | --- |
| Birthweight g | 3583.6 (705) | 3606.3 (688) | 3441.4 (800) |
| Macrosomia >=4kg | 59 (26.2) | 55 (28.4) | no observations |
| GROW centile | 82.1 (25.9) | 82.0 (25.9) | 82.0 (25.9) |
| INTERGROWTH centile | 85.7 (20.8) | 85.9 (20.6) | 84.3 (22.4) |
| WHO centile | 63.9 (32.0) | 64.4 (31.5) | 60.5 (35.2) |
| GROW >90^th^ centile | 140 (62.2) | 119 (61.3) | 20 (64.5) |
| INTERGROWTH >90^th^ centile | 150 (66.7) | 130 (67.0) | 20 (64.5) |
| WHO >90^th^ centile | 67 (29.8) | 59 (30.4) | 8 (25.8) |
| GROW <10^th^ centile | 5 (2.2) | 3 (1.55) | 1 (3.2) |
| INTERGROWTH <10^th^ centile | 3 (1.3) | 2 (1.0) | 1 (3.2) |
| WHO <10^th^ centile | 20 (8.9) | 15 (7.7) | 5 (16.1) |

**Table S3:** Comparison of GROW, INTERGROWTH and WHO growth standards in male and female infants. Data are presented as mean (SD) or n (%).

| BIRTHWEIGHT MEASURES | All infants  n=225 | Male infants  n=115 | Female infants  n=110 |
| --- | --- | --- | --- |
| Birthweight g | 3583.6 (705) | 3588.6 (695) | 3578.3 (719) |
| Macrosomia >=4kg | 59 (26.2) | 29 (25.2) | 30 (27.3) |
| GROW centile | 82.1 (25.9) | 81.9 (25.5) | 83.1 (25.2) |
| INTERGROWTH centile | 85.7 (20.8) | 85.9 (19.9) | 85.4 (21.8) |
| WHO centile | 63.9 (32.0) | 61.8 (31.6) | 66.1 (32.3) |
| GROW >90^th^ centile | 140 (62.2) | 72 (62.6) | 67 (60.9) |
| INTERGROWTH >90^th^ centile | 150 (66.7) | 78 (67.8) | 72 (65.5) |
| WHO >90^th^ centile | 67 (29.8) | 29 (25.2) | 38 (34.6) |
| GROW <10^th^ centile | 5 (2.2) | 1 (0.9) | 3 (2.7) |
| INTERGROWTH <10^th^ centile | 3 (1.3) | 1 (0.9) | 2 (1.8) |
| WHO <10^th^ centile | 20 (8.9) | 10 (8.7) | 10 (9.1) |

**Table S4:** Comparison of GROW, INTERGROWTH and WHO growth standards in infants delivered at term or preterm. Data are presented as mean (SD) or n (%).

| BIRTHWEIGHT MEASURES | All infants  n=225 | Term infants  n=136 | Preterm infants  n=89 |
| --- | --- | --- | --- |
| Birthweight g | 3583.6 (705) | 3787.0 (606) | 3272.7 (735) |
| Macrosomia >=4kg | 59 (26.2) | 45 (33.1) | 14 (15.7) |
| GROW centile | 82.1 (25.9) | 81.6 (25.0) | 84.0 (25.8) |
| INTERGROWTH centile | 85.7 (20.8) | 85.4 (20.8) | 86.2 (21.0) |
| WHO centile | 63.9 (32.0) | 73.3 (26.7) | 49.5 (34.0) |
| GROW >90^th^ centile | 140 (62.2) | 80 (58.8) | 59 (66.3) |
| INTERGROWTH >90^th^ centile | 150 (66.7) | 91 (66.9) | 59 (66.3) |
| WHO >90^th^ centile | 67 (29.8) | 52 (38.2) | 15 (16.9) |
| GROW <10^th^ centile | 5 (2.2) | 1 (0.7) | 3 (3.4) |
| INTERGROWTH <10^th^ centile | 3 (1.3) | 1 (0.7) | 2 (2.3) |
| WHO <10^th^ centile | 20 (8.9) | 4 (2.9) | 16 (18.0) |

**Supporting information**

**Appendix S1:** **CONCEPTT Collaborative Group** (listed according to recruitment numbers):

Cambridge University Hospitals NHS Foundation Trust, Cambridge, UK: Helen Murphy, Jeannie Grisoni,

Carolyn Byrne, Sandra Neoh, Katy Davenport, (43); Alberta Health Services, University of Calgary, Calgary,

Canada: Lois Donovan, Claire Gougeon, Carolyn Oldford, Catherine Young (39); King’s College Hospital,

London, UK: Stephanie Amiel, Katharine Hunt, Louisa Green, Helen Rogers, Benedetta Rossi (29); Mount

Sinai Hospital, Toronto, Canada: Denice Feig, Barbara Cleave, Michelle Strom (22); Hospital de la Santa

Creu i Sant Pau, Barcelona, Spain and CIBER‐BBN, Zaragoza, Spain: Rosa Corcoy, Alberto de Leiva, Juan

María Adelantado, Ana Isabel Chico, Diana Tundidor (22); The Ottawa Hospital General Campus, Ottawa,

Canada: Erin Keely, Janine Malcolm, Kathy Henry (15); Ipswich Hospital NHS Trust, Ipswich, UK: Damian

Morris, Gerry Rayman, Duncan Fowler, Susan Mitchell, Josephine Rosier (13); Norfolk and Norwich

University Hospital, Norwich, UK: Rosemary Temple, Jeremy Turner, Gioia Canciani, Niranjala

Hewapathirana, Leanne Piper (13); St. Joseph's Health Centre, London, Canada: Ruth McManus, Anne

Kudirka, Margaret Watson (13); Niguarda ca’ Granda Hospital, Milano, Italy: Matteo Bonomo, Basilio

Pintaudi, Federico Bertuzzi, Giuseppina Daniela Corica, Elena Mion (12); Sunnybrook Health Sciences

Centre, Toronto, Canada: Julia Lowe, Ilana Halperin, Anna Rogowsky, Sapida Adib (11); Glasgow Royal

Infirmary, Glasgow, UK: Robert Lindsay, David Carty, Isobel Crawford, Fiona Mackenzie, Therese McSorley

(10); McMaster University, Hamilton, Canada: John Booth, Natalia McInnes, Ada Smith, Irene Stanton,

Tracy Tazzeo (8); Centre hospitalier universitaire de Québec, Quebec City, Canada: John Weisnagel (6);

Queen’s Medical Centre, Nottingham, UK: Peter Mansell, Nia Jones, Gayna Babington, Dawn Spick (6);

Royal Victoria Infirmary, Newcastle Upon Tyne, Newcastle, UK: Malcolm MacDougall, Sharon Chilton, Terri

Cutts, Michelle Perkins (6); Leeds Teaching Hospitals NHS Trust, Leeds, UK: Eleanor Scott, Del Endersby

(6); Royal Infirmary of Edinburgh, Edinburgh, UK: Anna Dover, Frances Dougherty, Susan Johnston (6);

Sheffield Teaching Hospitals NHS Foundation Trust, Sheffield, UK: Simon Heller, Peter Novodorsky, Sue

Hudson, Chloe Nisbet (6); Izaak Walton Killam Health Sciences Centre (IWK), Halifax, Canada: Thomas

Ransom, Jill Coolen, Darlene Baxendale (5); University Hospital Southampton NHS Foundation Trust,

Southampton, UK: Richard Holt, Jane Forbes, Nicki Martin, Fiona Walbridge (6); Galway University

Hospitals, Galway, Ireland: Fidelma Dunne, Sharon Conway, Aoife Egan, Collette Kirwin (4); Central

Manchester University Hospitals NHS Foundation Trust, Manchester, UK: Michael Maresh, Gretta

Kearney, Juliet Morris, Susan Quinn (4); South Tees Hospitals, NHS Foundation Trust, Middlesbrough, UK:

Rudy Bilous, Rasha Mukhtar (4); Centre de Recherche du Centre Hospitalier de Université de Montréal

(CR‐CHUM), Montreal, Canada: Ariane Godbout, Sylvie Daigle (3); The Dudley Group NHS FT, Russells Hall

Hospital, Dudley, UK: Alexandra Lubina Solomon, Margaret Jackson, Emma Paul, Julie Taylor (3); Kingston

General Hospital, Queen’s University, Kingston, Canada: Robyn Houlden, Adriana Breen (3); Guys and St

Thomas’ NHS Foundation Trust, London, UK: Anita Banerjee, Anna Brackenridge, Annette Briley, Anna

Reid, Claire Singh (2); Royal University Hospital, Saskatoon, Canada: Jill Newstead‐Angel, Janet Baxter (2);

Grampian Diabetes Centre, Aberdeen, UK: Sam Philip, Martyna Chlost, Lynne Murray (2); William Sansum

Diabetes Center, Santa Barbara, USA: Kristin Castorino, Lois Jovanovic, Donna Frase (2). The Centre for

Clinical Trial Support (CCTS) at the Sunnybrook Research Institute, Toronto, Canada: Sonya Mergler,

Kathryn Mangoff, Johanna Sanchez, and Gail Klein. The Jaeb Center for Health Research, Tampa, USA:

Katrina Ruedy and Craig Kollman. Juvenile Diabetes Research Foundation (non‐clinical collaborators):

Olivia Lou and Marlon Pragnell.

**Appendix S2: Definitions of outcomes used in the CONCEPTT trial.**

**Birth Injury is defined by any of the following**:

- Spinal cord injury
- Basal skull fracture or depressed skull fracture
- Clavicular fracture
- Long bone fracture (humerus, radius, ulna, femur, tibia or fibula)
- Subdural or intracerebral hemorrhage of any kind [confirmed by cranial ultrasound, computerized tomography (CT) scan, or magnetic resonance imaging (MRI)])
- Peripheral Nerve Injury/Brachial Plexus

**Composite fetal outcome includes one or more of the following:**

- Pregnancy loss: miscarriage, stillbirth, neonatal death (death ≤28 days of life)
- Birth injury
- Neonatal hypoglycaemia
- Hyperbilirubinemia
- Respiratory distress
- High level neonatal care > 24 hours.

**Hyperbilirubinemia:**

Significant jaundice based on bilirubin levels requiring treatment with either phototherapy > 6 continuous hours, or an exchange transfusion, or receiving intravenous gamma globulin or requiring readmission into hospital during the first 7 days of life.

**Neonatal hypoglycemia:**

A plasma glucose <2.6 mmol/L on one or more occasions, starting at 30-60 minutes after birth, and necessitating intravenous dextrose, within the first 48 hours of life.

**Preterm birth:**

Preterm birth (<37 weeks and early preterm <34 weeks).

**Respiratory distress:**

Respiratory difficulties requiring any positive pressure ventilation ≥ 24 hours beyond resuscitation period (10 minutes), and/or given surfactant within 72 hours after birth.

**Shoulder Dystocia:**

Documentation of any shoulder dystocia in the delivery records, plus 3 or more of the following: McRoberts maneuver, suprapubic maneuver, episiotomy, delivery of the posterior arm, Woods maneuver, Rubins maneuver, All fours Gaskins maneuver, intentional fracture of the clavicle, and/or Zavenelli maneuver.
